# Photosynthesis Has Been Established Only Once—Evolution of Photosynthetic Reaction Center Proteins and Bacteriochlorophyll Biosynthesis

**DOI:** 10.3390/cimb48030306

**Published:** 2026-03-12

**Authors:** Johannes F. Imhoff, John A. Kyndt

**Affiliations:** 1 RD3 Marine Symbioses, GEOMAR Helmholtz Centre for Ocean Research Kiel, Wischhofstrasse 1-3, D-24148 Kiel, Germany; 2 College of Science and Technology, Bellevue University, Bellevue, NE 68005, USA

**Keywords:** photosynthesis evolution, photosynthetic reaction center, bacteriochlorophyll biosynthesis, cyanobacteria, phototrophic purple bacteria, Chloroflexi, Chlorobi, Heliobacteria, Chloracidobacteria

## Abstract

The first forms of photosynthetic life date back to more than 3.2–3.5 billion years ago. They performed photosynthesis in an anoxic world of the early earth and formed large mass accumulations on any kind of surface and in shallow waters. During evolution they diverged into several major phyla with differences in structure and function of the photosynthetic machinery including two different types of photosynthetic reaction centers. The combination of both of these types eventually led to the establishment of oxygenic photosynthesis present in cyanobacteria and chloroplast-containing plants. Common to all photosynthetic life is the presence of phylogenetically related reaction center proteins and of various chlorophyll molecules as mediators of light energy transformation into biochemical forms of energy. Therefore, we used phylogenetic analyses of reaction center proteins and representative enzymes of (bacterio)chlorophyll biosynthesis in addition to synteny of genome organization to unravel early divergencies of all known phyla of photosynthetic prokaryotes: Cyanobacteria, Chloroflexi, Chlorobi, Heliobacteria, Chloracidobacteria, and phototrophic purple Proteobacteria including *Gemmatimonas*. It was concluded that photosynthesis evolved only once, and all known forms diverged by various mechanisms from one primordial principal ancestor with properties resembling an ancestral cyanobacterium driving anoxygenic photosynthesis with a Type I photosystem and reduced inorganic electron donors.

## 1. Introduction

Life on earth as we know today essentially depends on the production of biomass by photosynthetic reactions using different forms of (bacterio)chlorophyll molecules. Therefore, understanding of the evolution of the process of photosynthesis and the biosynthesis of (bacterio)chlorophyll is of fundamental interest. It has gained much attention in the past decades. However, its evolutionary discussion still leaves several controversies about the order of events and whether photosynthesis has only evolved once or via multiple origins and pathways, as in, e.g., [1,2,3,4,5,6,7]. Controversial viewpoints on the origin of Type I and Type II photosynthetic reaction centers were discussed by Cardona [8] with the support of his own comparative analyses of photosynthetic reaction centers of all photosynthetic phyla.

Photosynthesis using (bacterio)chlorophyll-based processes is exclusively found in the Bacteria domain and originated in early forms of this domain. While the basic pathway for the biosynthesis of various bacteriochlorophyll molecules is shared by all photosynthetic bacteria, the structure of the photosynthetic apparatus shows significant variation in different lineages of these bacteria.

This variation includes the antenna structures, i.e., chlorosomes in Chlorobi and Chloroflexi, phycobilisomes in cyanobacteria, membrane-bound antenna complexes in Heliobacteria and special membrane intrusions in form of stacks, tubules or vesicles in phototrophic purple bacteria [9,10,11,12,13].

This variation also includes the molecular structure of the photosynthetic apparatus itself with a Type I photosystem in Heliobacteria, Chlorobi and Chloracidobacteria, a Type II photosystem in Proteobacteria and Chloroflexi and both Type I and Type II photosystems in the oxygenic phototrophic Cyanobacteria [14,15,16,17,18,19]. The key difference between Type I and Type II reaction centers is the terminal electron acceptor: the Type I photosynthetic apparatus uses iron–sulfur clusters for electron acceptance, while Type II uses a quinone. This divergence formed very early in evolution and represents a key step in the evolutionary history of phototrophy. It was suggested that the origin of the Type I and Type II reaction centers likely predates the diversification of most modern bacterial phyla, suggesting a very ancient evolutionary event [8]. While the Type I lineage was optimized for electron transfer under high-redox conditions by using an Fe-S cluster, the Type II lineage specialized in a quinone system as a cofactor [20].

Only the combination of Type I and Type II reaction centers allows for the performance of oxygenic photosynthesis using water as an electron donor and producing oxygen as the product. While the exact origin and the delineation of evolutionary lineages of oxygenic and anoxygenic photosynthesis in prokaryotes remain a matter of discussion, it is generally accepted that algae and higher plants acquired photosynthesis via endosymbiotic cyanobacteria-like bacteria that eventually became chloroplasts [21].

Photosynthetic reaction centers are the key element for photosynthesis and are therefore of utmost importance for establishment and evolution of the photosynthetic properties in different branches of phototrophic bacteria. Major currently known phylogenetic lineages are represented by photosynthetic reaction center proteins (psrc) of Type I in Cyanobacteria (PsaA and PsaB), Heliobacteria (PshA), Chlorobi (PscA) and Chloracidobacteria (PscA) and Type II in Cyanobacteria (PsbA and PsbD), Chloroflexi (PufL and PufM) and purple Proteobacteria including *Gemmatimonas* (PufL and PufM).

The number of available genomes of photosynthetic organisms has grown substantially in recent years and many more representative sequences are now available for each of these lineages, which makes an evolutionary analysis of the photosynthetic reaction centers more feasible and more robust than ever before.

The chlorophyll molecule with its various modifications is an essential part of photosynthetic energy generation in all bacterial lineages including Heliobacteria, Chlorobi, Chloroflexi, Chloracidobacteria, Cyanobacteria, purple bacteria and *Gemmatimonas*. The pathway of (bacterio)chlorophyll biosynthesis starts from protoporphyrin IX with the formation of Mg-protoporphyrin IX (by protoporphyrin IX Mg-chelatase, subunits BchDHI) and continues with the formation of the 6-monomethyl ester by Mg-protoporphyrin O-methyltransferase (BchM), followed by the formation of cyclic divinyl protochlorophyllide by Mg-protoporphyrin IX monomethyl ester oxidative cyclase (either aerobic, AcsF, or anaerobic, BchE). The C8-vinyl of divinyl protochlorophyllide may be reduced to form = protochlorophyllide. Finally, protochlorophyllide (or its divinyl form) is reduced by protochlorophyllide reductase to chlorophyllide and divinyl chlorophyllide respectively [22,23]. This central step in (bacterio)chlorophyll biosynthesis is catalyzed by protochlorophyllide reductase, a protein complex with the three subunits BchLNB, which is common to all photosynthetic bacteria and is involved in the biosynthesis of all bacteriochlorophyll and chlorophyll molecules [2,22]. Therefore, it is a perfect tool to study the phylogeny of (bacterio)chlorophyll biosynthesis. We chose this specific approach instead of comparing whole biosynthetic pathways in the different branches to unravel the biosynthesis of chlorophyll a compared to various bacteriochlorophyll molecules. The latter was not the aim of our present study and requires much more detailed analyses of the different biosynthetic pathways.

In order to trace back the evolution of photosynthesis, we compared the phylogenetic relationships of photosynthetic reaction center proteins involved in (bacterio)chlorophyll biosynthesis (protochlorophyllide reductase, BchN), the aerobic path of bchl biosynthesis (AcsF), and the synteny of genes in the neighborhood of *bchN*.

## 2. Materials and Methods

### 2.1. Phylogenetic Analysis

The sequences for the reaction center proteins, BchN, and AcsF were obtained from the respective genomes from the BV-BRC database [24]. In each case, a multiple-sequence alignment was performed using MUSCLE (version 3.8) [25], which uses seeded guide trees and Hidden Markov Model (HMM) profiles, specifically by perturbing HMM parameters and permuting guide trees to generate multiple-sequence alignments. The following parameters were used for the MUSCLE alignments. Gap penalties: gap open (−2.90), gap extend (0.00), hydrophobicity multiplier (1.20); cluster method (all iterations): UPGMA; min diag length lambda (24); max iterations (16). Table S1 contains information on genome sources used for the present study. The phylogenetic trees were generated in MEGA11 [26], which integrates Bayesian methods focused on efficiency and computing speed. The methods and parameters used are detailed in the following sections.

### 2.2. Reaction Center Protein Tree

For the construction of the reaction center phylogenetic tree, only single representatives of species genomes were used in the final analysis. In addition, the N-terminal antenna regions (300 aa) were removed from the Type I sequences to assure that all sequences used were of comparable length (from 275 to 450 aa) and only the core protein sequences were used for comparison. To determine the best evolutionary model, we ran a “find best protein model” analysis for our dataset in MEGA11, which is an analysis that compares the available phylogenetic models and variations in MEGA and uses Bayesian Information Criteria (BIC) as the primary metrics for selecting the best-fit model. Four of the six top models were variations of the Le_Gascuel_2008 (with Gamma distribution) model and the other two were WAG (Whelan and Goldman)-model-based. We therefore decided to infer the evolutionary history by using the Maximum Likelihood method and Le_Gascuel_2008 model [27]. The tree with the highest log likelihood (−21,201.58) is shown. The percentage of trees in which the associated taxa clustered together is shown next to the branches. The initial tree(s) for the heuristic search was obtained automatically by applying Neighbor-Join and BioNJ algorithms to a matrix of pairwise distances estimated using the JTT model, which is a well-characterized standard substitution model with a Bayesian framework, and then selecting the topology with superior log likelihood value. A discrete Gamma distribution was used to model evolutionary rate differences among sites (5 categories (+*G*, parameter = 4.9430)). The rate variation model allowed for some sites to be evolutionarily invariable ([+*I*], 0.00% sites). This analysis involved 141 amino acid sequences. All positions with less than 90% site coverage were eliminated, i.e., fewer than 10% alignment gaps, missing data, and ambiguous bases were allowed at any position (partial-deletion option). There were a total of 240 positions in the final dataset. Bootstrap values were generated from 200 bootstrapping rounds. Evolutionary analyses were conducted in MEGA11. iTOL (version 7.5) was used to draw the phylogenetic tree [28]. The tree was midpoint-rooted in iTOL.

### 2.3. BChN Tree

The evolutionary history was inferred by using the Maximum Likelihood method and Le_Gascuel_2008 model [27]. The tree with the highest log likelihood (−22,682.62) is shown. The percentage of trees in which the associated taxa clustered together is shown next to the branches. The initial tree(s) for the heuristic search was obtained automatically by applying Neighbor-Join and BioNJ algorithms to a matrix of pairwise distances estimated using the JTT model, which is a well-characterized standard substitution model with a Bayesian framework, and then selecting the topology with superior log likelihood value. A discrete Gamma distribution was used to model evolutionary rate differences among sites (5 categories (+*G*, parameter = 0.8134)). The rate variation model allowed for some sites to be evolutionarily invariable ([+*I*], 2.14% sites). This analysis involved 99 amino acid sequences. All positions with less than 90% site coverage were eliminated, i.e., fewer than 10% alignment gaps, missing data, and ambiguous bases were allowed at any position (partial-deletion option). There were a total of 386 positions in the final dataset. Bootstrap values were generated from 200 bootstrapping rounds. Evolutionary analyses were conducted in MEGA11. iTOL (version 7.5) was used to draw the phylogenetic tree [28]. The tree was midpoint-rooted in iTOL.

### 2.4. AcsF Tree

The evolutionary history was inferred by using the Maximum Likelihood method and Le_Gascuel_2008 model [27]. The tree with the highest log likelihood (−13,964.56) is shown. The percentage of trees in which the associated taxa clustered together is shown next to the branches. The initial tree(s) for the heuristic search was obtained automatically by applying Neighbor-Join and BioNJ algorithms to a matrix of pairwise distances estimated using the JTT model, which is a well-characterized standard substitution model with a Bayesian framework, and then selecting the topology with superior log likelihood value. A discrete Gamma distribution was used to model evolutionary rate differences among sites (5 categories (+*G*, parameter = 1.1171)). The rate variation model allowed for some sites to be evolutionarily invariable ([+*I*], 8.82% sites). This analysis involved 42 amino acid sequences. All positions with less than 90% site coverage were eliminated, i.e., fewer than 10% alignment gaps, missing data, and ambiguous bases were allowed at any position (partial-deletion option). There were a total of 337 positions in the final dataset. Bootstrap values were generated from 200 bootstrapping rounds. Evolutionary analyses were conducted in MEGA11 [26]. iTOL (version 7.5) was used to draw the phylogenetic tree [28]. The tree was midpoint-rooted in iTOL.

### 2.5. Synteny Analysis

Synteny analysis was performed in BV-BRC, which uses the Proteome Comparison tool, which is based on the RASTk (Rapid Annotation using Subsystem Technology) toolset. Comparative genome regions were generated using global PGFam families to determine a set of genes that match a focus gene. The gene set was compared to the focus gene using BLAST (version 2.16.0) and sorted by BLAST scores within BV-BRC [24]. The Compare Region Viewer in BV-BRC displays the focus gene along with the other genes in the same family and their flanking regions in their genomes. The *bchN* gene was used as a focus gene to analyze synteny of the photosynthetic and bacteriochlorophyll gene clusters.

## 3. Results

### 3.1. Evolution of Photosynthetic Reaction Center Proteins

**Figure 1:**
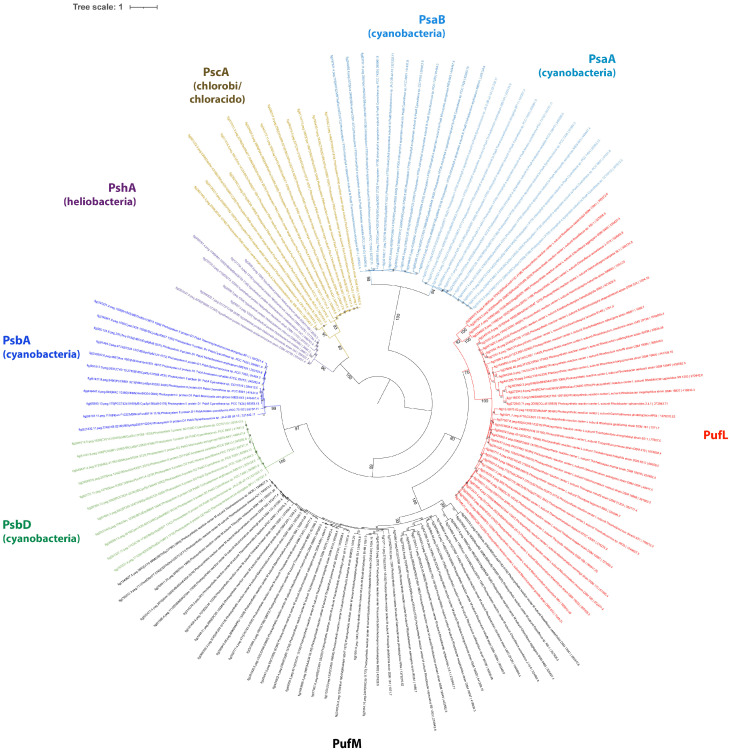
Phylogenetic tree using the Type I and Type II reaction center protein sequences. Sequences were translated sequences derived from whole-genome sequences. Accession numbers are indicated for each sequence. The phylogenetic tree was calculated in MEGA11 [26] using the Maximum Likelihood method and Le_Gascuel model [27] with Gamma distribution and allowing for some sites to be evolutionarily invariable (LG + G + I method) and iTOL was used to draw the phylogenetic tree [28]. Bootstrap values were generated from 200 bootstrapping rounds. The tree was midpoint-rooted and the different-colored clades contain sequences from Cyanobacteria, Heliobacteria, Chlorobi, Chloroacidobacteria, Chloroflexi and purple bacteria.

From the sequence information and their phylogenetic relationships, it is apparent that all reaction center proteins are distantly related to each other (Figure 1). A rectangular version of this phylogenetic tree can be found in Figure S1.

As far as we can delineate from the relationship between the reaction center protein sequences, both Type I and Type II reaction center proteins represent separate evolutionary lines with a most ancient common ancestor (Figure 1), which predates the first Type I and Type II photosynthetic reaction centers that we know. From this relationship, the following conclusion can be drawn.

The evolution of photosynthetic reaction centers started from a primordial homodimeric ancestor that presumably predated the first homodimeric Type I reaction center proteins that evolved into the Type I reaction centers of Heliobacteria, Chlorobi, Chloracidobacteria and Cyanobacteria. Until today homodimeric reaction centers are present in Heliobacteria (PshA), Chlorobi and Chloracidobacteria (PscA). The sequences of these reaction centers form a cluster clearly separate from those of Cyanobacteria and their divergence occurred very early in photosynthesis evolution.

The separation of Type I and Type II reaction center proteins was also a very early event in evolution. In the branch of Type II reaction center proteins, separate and early diverging lineages are formed by Cyanobacteria (PsbA/D) on one hand and Chloroflexi, proteobacterial phototrophic purple bacteria and *Gemmatimonas* (PufL/M) on the other hand. The phylogenetic relations suggest early divergence and independent evolution of the cyanobacterial Type II reaction center proteins from those of Chloroflexi and phototrophic purple bacteria.

While homodimeric reaction centers have survived until today only in the three mentioned lineages with Type I reaction centers, in all other lineages a heterodimeric system has evolved. Quite remarkable, all heterodimers of PS I and PS II in Cyanobacteria (PsaA/B, PsbA/D) and of Type II reaction centers in Chloroflexi, Proteobacteria and *Gemmatimonas* (PufL/M) are closely related to their direct counterpart, which strongly suggests independent and parallel events of gene duplication as the mechanisms for introducing the heterodimer into the reaction centers. Parallel events of gene duplication in all branches happened at a much later time as compared to the separation of the homodimeric reaction center proteins. Therefore, the first photosynthetic reaction centers were homodimeric at their origin and the presently known heterodimeric reaction centers of both Type I and Type II were formed much later.

Indeed, the Type II photosynthetic reaction center proteins revealed a common early ancestor with ancestral homodimeric Type I reaction center proteins, in particular those of cyanobacterial photosynthetic reaction centers. While they were supposedly homodimeric at the beginning as well, today, they are represented by the heterodimeric photosynthetic reaction centers of Chloroflexi and phototrophic purple bacteria (PufL/M) and those of Cyanobacteria (PsbA/D). Both Type II reaction centers (PufL/M and PsbA/D) had a common origin but evolved separately and independently from each other, and much later formed heterodimeric reaction centers. Apparently, none of the homodimeric forms of these reaction centers survived during evolutionary times and the precursor of photosynthetic reaction centers from Cyanobacteria on one hand and from Chloroflexi and purple bacteria on the other hand already had a heterodimeric reaction center. In this context it is interesting that in both the PufL and PufM branches, the Chloroflexi cluster together with the purple bacteria and show the deepest divergence among these genes (Figure 1). This indicates that photosynthetic reaction centers of Chloroflexi are the most ancient representative of the PufL/PufM type of reaction center and the closest of this branch to a common ancestor with the PS II of Cyanobacteria.

The only viable conclusion that we can draw from this is that photosynthesis has been established once and all current forms of photosynthetic reaction center proteins have a single origin. Separate lines of evolution formed the Type I photosynthetic reaction centers in Heliobacteria, Chlorobi and Chloracidobacteria and developed to the PS I in Cyanobacteria. Ancestral forms of the Type I photosynthetic reaction center also represent the primordial ancestor of all Type II photosynthetic reaction centers, which may have originated by gene duplication in an ancient anoxygenic ancestor of cyanobacteria and Chloroflexi.

### 3.2. Bacteriochlorophyll Biosynthesis—BchN Phylogeny

**Figure 2:**
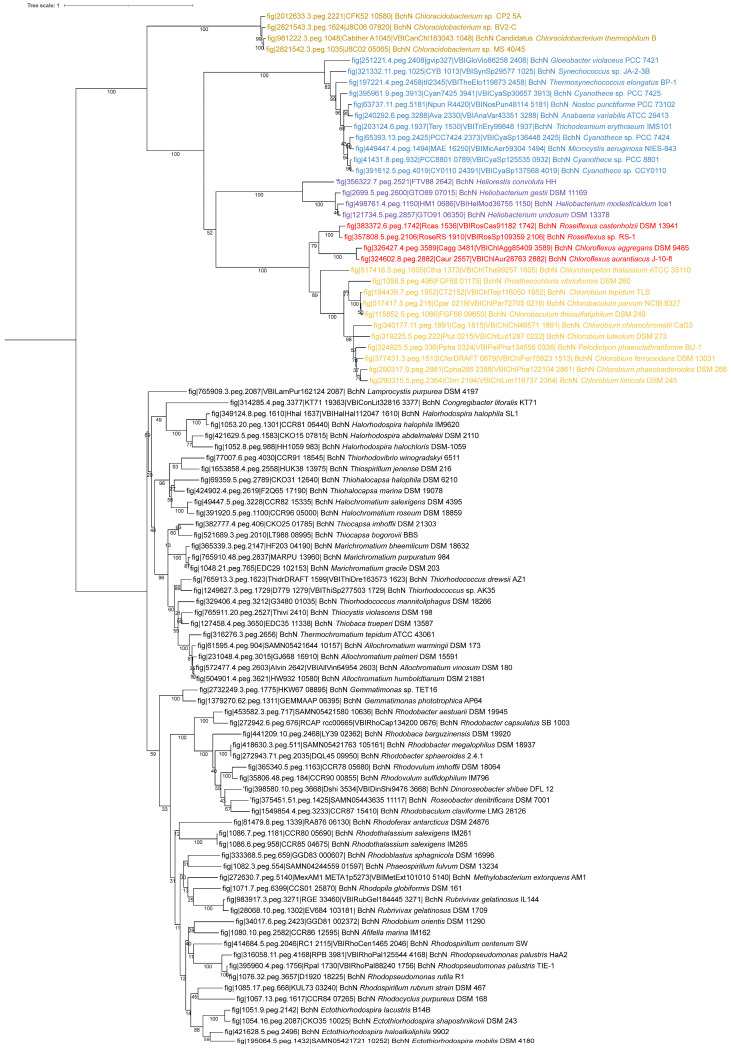
Phylogenetic tree using the BchN protein sequences. Sequences were translated sequences derived from whole-genome sequences. Accession numbers are indicated for each sequence. The phylogenetic tree was calculated in MEGA11 [26] using the Maximum Likelihood method and Le_Gascuel model [27] with Gamma distribution and allowing for some sites to be evolutionarily invariable (LG + G + I method), and iTOL was used to draw the phylogenetic tree [28]. Bootstrap values were generated from 200 bootstrapping rounds. The tree was midpoint rooted and the different colored clades contain sequences from Chloroacidobacteria, Heliobacteria, Cyanobacteria, Chloroflexi, Chlorobi, and purple bacteria.

Representative for the biosynthesis of bacteriochlorophyll molecules, the sequences of BchN, which is a central part of protochlorophyllide reductase BchLNB, have been compared (Figure 2). A basic phylogenetic divergence of BchN distinguishes two major branches with distinct lineages of Chloracidobacteria, Cyanobacteria and Heliobacteria, Chlorobi and Chloroflexi in one and the phototrophic Proteobacteria including *Gemmatimonas* in another branch. All major lineages appear to be monophyletic. The sequences of Chloracidobacteria are most distant to all others. Lineages of Chlorobi and Chloroflexi are more closely related than all others. Within the Chloroflexi lineage, *Chloroflexus* and *Roseiflexus* form two clearly distinct lines. Thus, BchN of all bacteria with Type I photosynthetic reaction centers, including Cyanobacteria and Chloroflexi, has a common ancestor, which separates this lineage from the phototrophic purple bacteria including *Gemmatimonas*. The phylogenetic relationships of BchN are quite similar to those of other genes of chlorophyll biosynthesis (BchXYZ) and of 16S rRNA [29]. The phylogeny of photosynthetic reaction centers differs in one important point, the position of Chloroflexi. The presence of a Type II photosynthetic reaction center and the phylogenies of reaction center proteins PufLM clearly separate Chloroflexi from Type I photosynthetic reaction center lineages and place them at the bottom of a major lineage with Type II photosynthetic reaction centers together with phototrophic purple bacteria, but apart from the lineage of cyanobacterial Type II reaction centers.

### 3.3. Presence and Phylogeny of AcsF

The onset of oxygenic photosynthesis dramatically changed conditions of bacteriochlorophyll biosynthesis, which basically was quite oxygen sensitive. As a consequence, phototrophic bacteria needed to escape oxygen in the environment, protect bacteriochlorophyll biosynthesis from oxygen or replace the oxygen-sensitive Mg-protoporphyrin IX monomethyl ester oxidative cyclase (BchE) by an aerobic enzyme AcsF. Therefore, the presence and phylogeny of this enzyme are of particular interest to understanding the evolution of photosynthesis.

**Figure 3:**
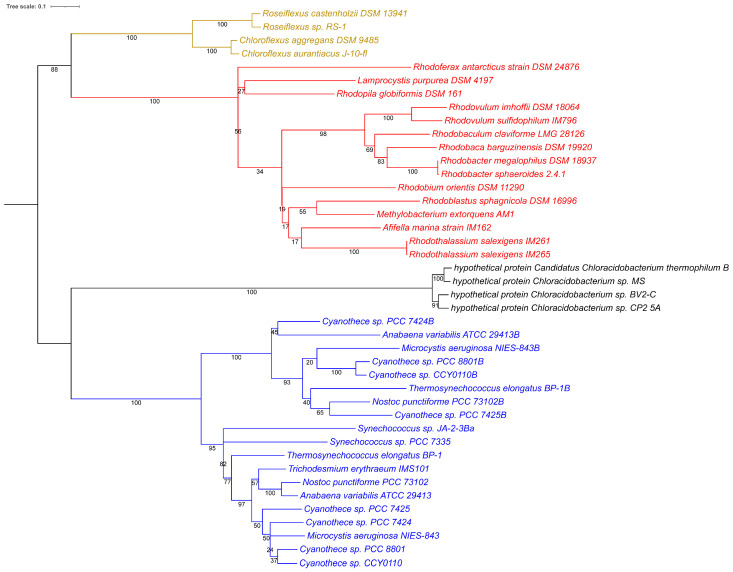
Phylogenetic tree using the AcsF protein sequences. Sequences were translated sequences derived from whole-genome sequences. Accession numbers are indicated for each sequence. The phylogenetic tree was calculated in MEGA11 [26] using the Maximum Likelihood method and Le_Gascuel model [27] with Gamma distribution and allowing for some sites to be evolutionarily invariable (LG + G + I method), and iTOL was used to draw the phylogenetic tree [28]. Bootstrap values were generated from 200 bootstrapping rounds. The tree was midpoint rooted and the different colored clades contain sequences from Cyanobacteria, Chloroflexi, and purple bacteria.

In addition to Cyanobacteria, Chloroflexi and Chloracidobacteria, a number of phototrophic purple bacteria have the possibility of bacteriochlorophyll biosynthesis in the presence of oxygen using the aerobic Mg-protoporphyrin IX monomethyl ester oxidative cyclase AcsF. This ability is absent from Heliobacteria and Chlorobi but also from many phototrophic purple bacteria, which may have lost the enzyme during evolution. The phylogeny of AcsF indicates that a common precursor of AcsF existed prior to the separate evolution in Cyanobacteria in one and in Chloroflexi and purple bacteria in the other lineage. An early separation occurred of the major AcsF lineages (Figure 3).

### 3.4. Genome Organization—Synteny

**Figure 4:**
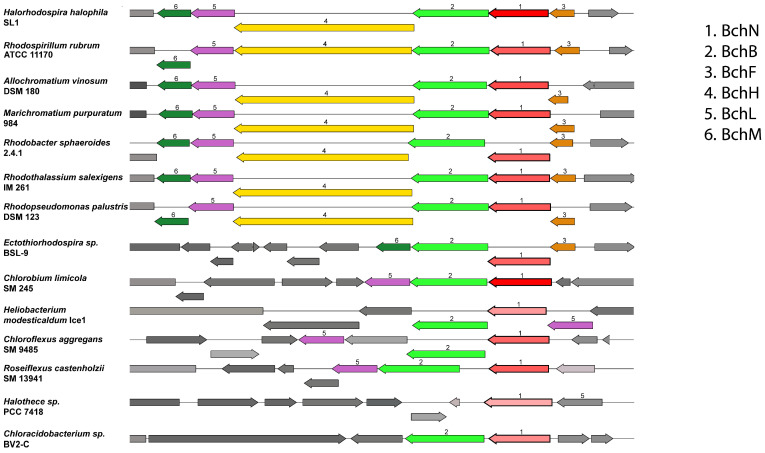
Synteny of the *bchN* genomic region in representative genomes from each of the bacterial groups: purple bacteria, Chlorobi, Heliobacteria, Chloroflexi and Cyanobacteria. Synteny plots were generated in BV-BRC, which uses the Proteome Comparison tool [24]. Genes are colored based on their family membership. Only genes relevant to the bacteriochlorophyll synthesis are labeled for clarity.

The organization of genes related to photosynthesis in smaller or larger gene clusters and their distribution among the genome varies greatly within the different groups of phototrophic bacteria. The major phylogenetic groups can be differentiated by significant and stable differences in the genomic organization of genes related to photosynthesis and bacteriochlorophyll biosynthesis. As an example, Figure 4 shows a general overview of the synteny around the *bchN* gene in representative genomes of the major phylogenetic groups. A more comprehensive overview of gene synteny comprising several genomes from each major group can be found in Supplemental Figure S2.

The lack of cluster formation is most significant within Cyanobacteria, but also obvious in Chlorobi, Chloroflexi and *Chloracidobacterium* (Figure 4 and Figure S2). In these groups, photosynthetic genes occur as single separated genes or only small clusters at different locations within the genome. We assume that progress in evolution has given rise to the formation of extended gene clusters and increase in organization, which apparently is a phenomenon common to evolutionary processes [30]. With this assumption we must conclude that early ancestors of photosynthetic bacteria had no or only poorly organized gene clusters for photosynthesis. The same holds for the degree of regulation (and facilitated assembly) of the biosynthesis and function of the photosynthetic apparatus. Obviously, the genomic organization of photosynthesis genes in the mentioned groups has maintained a more-or-less ancient situation.

In contrast, genomic organization in purple bacteria is the most advanced and, in these bacteria, photosynthetic genes occur in a large cluster including genes coding for biosynthesis of reaction center proteins, bacteriochlorophylls, carotenoids and their regulation.

This is highlighted by focusing on the neighborhood of *bchN*. In phototrophic purple bacteria, which show the highest degree of cluster formation, this gene is included in a *bchFNBHLM* cluster, present in most phototrophic purple bacteria of Gammaproteobacteria (Halorhodospiraceae and Chromatiaceae), Alpha- and Betaproteobacteria (Figure 4) and *Gemmatimonas*. A modification of this strongly conserved cluster is seen in *Ectothiorhodospira* species with a *bchFNB-M* cluster and separate locations for *bchL* and *bchH* genes. In contrast, *bchN* is included in a much smaller *bchLBN* cluster in Chlorobi and in a *bchLNB* cluster in Heliobacteria. The lowest level of cluster formation is found in Cyanobacteria with separate gene loci for *bchLN* and *bchB*, e.g., in *Halothece* species PCC7418 and in *Chloracidobacterium* with *bchBN*. In *Chloroflexus* separate gene locations are seen for *bchNB-L*, *bchM* and *bchH*, while an *NBLM-H* cluster is present in *Roseiflexus* (Figure 4).

The low order in the structure of the gene clusters suggests that Chloroflexi have maintained most ancient properties in the branch with Type II photosynthetic reaction centers, which excludes gain of photosynthesis by horizontal gene transfer. Clear differences occur between *Chloroflexus* and *Roseiflexus* branches with somewhat increased cluster formation of genes in *Roseiflexus*. In this respect, *Roseiflexus* appears more similar to proteobacterial purple bacteria than *Chloroflexus*. It is interesting to note that *Roseiflexus* lacks chlorosomes and possibly has lost these organelles during evolution. In addition, the *pufBALMC* cluster is present in *Roseiflexus* in the same order as in the purple bacteria, but different from the *puf*BAC cluster in *Chloroflexus*. These properties place the photosynthetic properties of *Roseiflexus* in closer relations to those of phototrophic purple Proteobacteria.

## 4. Discussion

### 4.1. Phylogeny of Reaction Centers

According to the available sequence information (Figure 1 and Figure S1), the first phototrophic bacterium was most likely using a precursor of a Type I homodimeric reaction center from which those of Cyanobacteria, Heliobacteria, Chlorobi and Chloracidobacteria evolved. This primordial ancestor existed in an anoxic and sulfidic strongly reducing environment that prevailed for more than a billion years on the early earth. It is most likely that under these conditions, sulfide and possibly other reduced inorganic sources (e.g., hydrogen and ferrous iron) served as photosynthetic electron donors. The fact that sulfide supports CO_2_ assimilation in *Oscillatoria limnetica* in the presence of DCMU (an inhibitor of photosystem II) proved that photosystem I is involved in light reactions and that sulfide can act as a photosynthetic electron donor in present-day Cyanobacteria [31,32]. In addition, photoassimilation of CO_2_ and photosynthesis driven only by photosystem I was demonstrated in *Oscillatoria limnetica* with sulfide as the electron donor (oxidation to elemental sulfur) [31,32]. The potential of sulfide-dependent anoxygenic photosynthesis is apparently widely distributed among Cyanobacteria until today [33,34,35,36,37]. It is therefore a realistic scenario that a primordial most ancient ancestor of cyanobacteria used a Type I homodimeric reaction center and sulfide as the electron donor for anoxygenic photosynthesis. Only under the highly reducing environmental conditions under which early photosynthesis was established could photosynthetic reactions prevailing in Type I photosynthetic reaction centers have evolved. In accordance with this and with the phylogeny of reaction center proteins, the existence of Type I reaction center proteins predates that of Type II reaction center proteins and anoxygenic photosynthesis of the primordial phototrophic bacteria may have used a precursor of a Type I photosynthetic reaction center.

During geological time scales, early ancestors of Cyanobacteria (and Chloroflexi) with Type I photosynthetic reaction centers gained a second photosystem possibly by gene duplication. This view is supported by a remarkably conserved three-dimensional protein and cofactor structure of Type I and Type II reaction centers [8,38,39]. Over time this system developed into the Type II photosynthetic reaction center, which is found in phototrophic purple bacteria and Chloroflexi on one hand and formed the water-splitting enzyme as it is found in present-day Cyanobacteria on the other hand. In accord with this assumption is the finding that the antenna domain of Type I reaction center proteins is homologous to core antenna of PS II reaction centers in Cyanobacteria, and it was suggested that core antenna proteins of PS II (PsbC and PscB) of Cyanobacteria arose from duplications of antenna domains of PS I [40,41]. In this context it should be noted that Type I reaction centers have a long core protein that includes an antenna domain, while Type II reaction centers have a short core protein that does not contain an antenna domain but is associated with antenna proteins that perform a function analogous to the antenna domain of Type I core proteins [42]. Note that the additional antenna region in the Type I sequences was ignored for our PS phylogenetic analyses to allow for a more uniform length distribution and to assure that only the core domain regions were compared. The presence of this longer antenna domain in Type I reaction centers is an additional indication that PS II has evolved from an ancient precursor of a Type I photosystem, most likely by gene duplication in ancestral relatives of cyanobacteria.

### 4.2. Relation Between Chlorobi and Chloroflexi

Photosynthesis of green sulfur bacteria of the Chlorobi phylum and the green nonsulfur bacteria of the Chloroflexi is phylogenetically closely related. This is reflected in the relation of phylogeny of bacteriochlorophyllide, a reductase BchXYZ involved in bacteriochlorophyll synthesis [29], and protochlorophyllide reductase subunit BchN (Figure 2). In both groups the three subunits of this protein L, N and B are included in one gene cluster (LBN in Chlorobi, NB-L in *Chloroflexus* and NBLM in *Roseiflexus*), which differentiates the two groups from Cyanobacteria (LN), purple bacteria (FNBHLM), Heliobacteria (LNB) and Chloracidobacteria (BN). Unique to both Chlorobi and Chloroflexi is a *bchFCX* gene cluster. They also share the presence of special light-harvesting organelles, the chlorosomes, which are most powerful in collecting minute amounts of light for photosynthesis, except for the *Roseiflexus* lineage, which may have lost these organelles. The most significant difference is the presence of a Type II reaction center in Chloroflexi, though all of these properties place Chloroflexi close to Chlorobi and other lineages with a Type I photosynthetic reaction center.

As the Cyanobacteria and other photosynthetic lineages related to Chloroflexi have a Type I photosynthetic reaction center, it is reasonable to assume that ancestors of Chloroflexi might have expressed a Type I photosynthetic reaction center at their origin as well. They could have gained a Type II reaction center and lost the Type I reaction center.

As it is likely that the Type II reaction center of Cyanobacteria originated by gene duplication from a primordial Type I reaction center [41] and their Type II reaction center proteins are the most similar to those of Chloroflexi (Figure 1), it is reasonable to assume that Type II photosynthetic reaction centers originated from gene duplication in a common ancestor of both Chloroflexi and Cyanobacteria, which could very well represent the primary ancestor of all Type II photosynthetic reaction centers.

In the cyanobacterial branch, the Type I photosynthetic reaction center was maintained and the duplicated form developed into the water-splitting PS II of oxygenic phototrophs. In the branch of Chloroflexi the Type I photosynthetic reaction center was lost and Type II developed into an independent photosystem as presently known from Chloroflexi and purple bacteria. The phylogenetic relations suggest that common ancestors of Chloroflexi and Cyanobacteria represent the first bacteria with a Type II photosynthetic reaction center and there is no known bacterium or lineage of bacteria that at that time could have served as a donor for this type of reaction center by any kind of transfer process. The photosynthetic reaction centers of phototrophic purple bacteria share a common origin with those of Type II reaction centers of Chloroflexi and evolved after loss of Type I photosynthetic reaction centers and establishment of heterodimers.

### 4.3. Gain or Loss

During evolution, both gains and losses of photosynthesis genes may have occurred along geological time scales. Losses usually cannot be traced while gains may occur via horizonal transfer or gene duplication and show sequence similarities with their parent genes. While horizonal transfer has often been used to explain phylogenetic discrepancies, gene duplication is not consequently considered as a possible mechanism. Genomes of extant cyanobacteria contain multiple gene copies encoding reaction center proteins [8] and it is reasonable to assume that this also was a property of ancient ancestors of these bacteria and a possible driving force for the evolution of photosynthetic reaction centers. Different lines of evolution of gene multiplicates could, e.g., be the basis for different types of photosynthetic reaction centers deriving from the same origin.

Although several examples have been reported for horizontal gene transfer among phototrophic bacteria, it is not known whether and to what extent such mechanisms were available in the early days of evolution, and it appears almost impossible to trace such events to these early developments. Nevertheless, in a few cases horizontal gene transfer has been clearly demonstrated. Photosynthetic genes can be located on bacterial plasmids [43] and were also repeatedly found in viruses of Cyanobacteria [30,44,45,46], which are potential agents of gene transfer between species. The most popular example of transfer of complete photosynthesis gene clusters is *Gemmatimonas phototrophica* [47] and lateral gene transfer has also been postulated to explain inconsistencies in the phylogeny of different genes within the closely related group of phototrophic Rhodobacteraceae [43].

Photosynthesis genes are considered to have been scattered around the genome of ancient photosynthetic lineages, and this is still the case in the present-day genomes, most pronounced in Cyanobacteria, but also in Chlorobi, Chloracidobacteria and Chloroflexi, with photosynthesis genes located at several different positions within the genome. The most advanced organization is found in phototrophic Proteobacteria, which have extended photosynthesis gene clusters. Heliobacteria have a few small clusters of photosynthesis genes. The existence of a scattered genomic distribution of photosynthesis genes makes it quite unlikely that larger parts of its biosynthetic pathways were horizontally transferred and that non-phototrophic bacteria gained photosynthesis via horizontal gene transfer in ancient times of evolution. This still holds for present-day representatives of these groups. In consequence, the loss of photosynthetic capabilities during evolution was much more likely than the gain of this property by horizontal gene transfer.

The major challenge to this view is the presence of Type II photosynthetic reaction centers in Chloroflexi, which in other terms fit into the group of phototrophic bacteria with Type I photosynthetic reaction centers. Taking into account that the Type II photosynthetic reaction centers of Cyanobacteria most likely arose by gene duplication from a Type I precursor and that these sequences are closer to those of Chloroflexi than to others, it is reasonable to assume that this gene duplication happened in a common ancestor of the two groups of bacteria prior to their separation. The separation of the two lines of Type II photosynthetic reaction centers of Cyanobacteria and Chloroflexi already occurred at a very early stage during the evolution of Type II photosystems, leading to the different types of photosynthetic reaction centers in Chloroflexi and purple bacteria (PufL/M) and Cyanobacteria (PsbA/D).

### 4.4. Geological Records

Much of the information on the evidence of early photosynthesis originates from ancient geological records. Although numerous attempts of correlations between geological data and photosynthesis evolution exist, we will focus on a few special aspects here. This includes microbial mats of filamentous bacteria, the carbon isotope ratio and the presence of special lipid components. In general, assumptions are made based on the properties of present-day representatives. However, the projection towards the early times of evolution and their interpretation as presence of cyanobacteria and oxygenic photosynthesis may not always be straightforward, and sometimes may even be misleading.

From the occurrence of stromatolite-like structures in ancient geological formations, it was suggested that some filamentous forms of phototrophic life were already present 3.2–3.5 billion years ago [3]. Such microbial mats could have been formed by ancient representatives of primordial ancestors of Cyanobacteria or Chloroflexi. In both groups filamentous forms still exist today. However, oxygenic photosynthesis certainly was not possible at that time.

Geological evidence from carbon isotope studies at the Buck Reef Chert along the South African coast indicated that partially filamentous microbial communities existed ca. 3.4 billion years ago and CO_2_ fixation took place by the Calvin Cycle [48] and this was taken as evidence for the activity of primordial kinds of cyanobacteria. However, it has been demonstrated that some present-day Chloroflexi, i.e., *Oscillochloris trichoides*, can also use the Calvin Cycle in contrast to most known species that perform an alternative route [49]. Therefore, in projection to early ancestors, the possibility of CO_2_ fixation by common ancestors of both Cyanobacteria and Chloroflexi appears reasonable.

Also, the presence of 2-methylbacteriohopanepolyol and its degradation product 2-methylhopane in 2.5 to 2.7-billion-year-old geological settings was taken as evidence for the presence of oxygen and active oxygenic photosynthesis by Cyanobacteria and their ancient ancestors, because the known biosynthetic route requires molecular oxygen [50]. This conclusion could be misleading [50,51], because more recent studies indicate that other microorganisms also produce these lipids and that 2-methylbacteriohopanepolyols can be produced by anoxygenic phototrophic Proteobacteria in the absence of oxygen [52,53]. Nevertheless, it appears reasonable that oxygenic photosynthesis was already active at that time and in some ecological niches oxygen could have already locally accumulated several hundreds of thousands of years before the Great Oxygen Event.

Much evidence has accumulated that oxygenic photosynthesis was established, and free oxygen accumulated in the atmosphere, approx. 2.4 billion years ago [54]. In the literature this time often is dated as the Great Oxygen Event (GOE). Consistent with the appearance of free oxygen is the observation of massive ferric iron deposition in banded iron sediment formations 2.5 billion years ago [3]. Banded iron formation can form only when abundant dissolved ferrous iron is transported into depositional basins, and an oxygenated ocean blocks this transport by oxidizing the iron to insoluble ferric iron compounds. Extensive deposits of banded iron formations are found around the world. After peaking at about 2.5 billion years, they largely disappeared from the geological record until 1.85 billion years ago [55]. The end of the deposition of banded iron formation at 1.85 billion years ago is interpreted as disappearance of dissolved iron (Fe-II), marking the oxygenation of the deep ocean [50]. As it was suggested that during evolution of bacterial species, major lineages of Hydrobacteria (including Chlorobi and Proteobacteria) and Terrabacteria (including Cyanobacteria, Chloroflexi and Heliobacteria) diverged around 3.2 billion years ago [56,57], we suppose that all major lines of phototrophic bacteria were established prior to 3.2 or even 3.5 billion years ago. This is in accord with the presence of filamentous phototrophic bacteria approx. 3.5 billion years ago [3].

These observations give a time of more than 1 billion years for the evolution of photosynthetic bacteria, from the presence of filamentous microbial mats at approx. 3.5 billion years ago to the first accumulation of free oxygen in the atmosphere at around 2.5 billion years ago, i.e., from anoxygenic photosynthesis of primordial ancestors of Cyanobacteria and Chloroflexi until the established oxygenic photosynthesis of Cyanobacteria.

### 4.5. The Scenario

The phylogenetic data from photosynthetic reaction center proteins and bacteriochlorophyll biosynthesis (Figure 1, Figure 2 and Figure 3) clearly demonstrate that evolutionary precursors of homodimeric Type I photosynthetic reaction centers were the first and invented by common primordial ancestors of Cyanobacteria, Chloroflexi, Heliobacteria, Chloracidobacteria, and Chlorobi. They have been retained through further evolution as such in the last three groups, which are restricted to very special ecological niches in present-day environments and thereby are limited in further evolutionary development. Later (but still more than 3.2 billion years ago, prior to the presumed separation of Terrabacteria and Hydrobacteria), in a primordial ancestor of Cyanobacteria, Chloroflexi and Proteobacteria, the duplication of Type I reaction center genes first of all may have initiated a primordial Type II reaction center. The current repertoire of phototrophic bacteria suggests the following developments of Type II photosynthetic reaction centers:

• In the cyanobacterial lineage the adaptation and coordinated function of the primordial Type II photosynthetic reaction center led to the transformation into a water-splitting system as the fundamentally significant advantage to promote oxygenic photosynthesis. In consequence, the relatives lacking this property were outcompeted and ultimately became extinct.
• In ancestral precursors of Chloroflexi, which perform an anoxygenic form of photosynthesis, the loss of Type I photosynthesis and parallel development of the primordial Type II photosynthetic reaction center into a functional Type II photosynthetic reaction center without the capacity to use water as the electron donor led to a homodimeric Type II reaction center.
• Independent duplication of the reaction center genes much later led to Type II heterodimeric reaction centers (*pufL/M*) in Chloroflexi and phototrophic purple bacteria and of Type I and Type II heterodimeric reaction centers in Cyanobacteria (*psaA/B*, *psbA/D*). These gene duplications were apparently of significant advantage and led to the extinction of parent strains lacking the gene duplicate, in this example those with homodimeric photosynthetic reaction centers. As a consequence we do not see these in the current repertoire of living phototrophs.
• The Type II photosynthetic reaction centers as we see today within phototrophic purple bacteria and Chloroflexi originated from an intermediate form that had lost the Type I photosynthetic reaction center but already had acquired a heterodimeric complex.
• The absence of any known anoxygenic phototroph that uses both a Type I and a Type II photosystem is paradoxical. Current models cannot explain why a two-photosystem anoxygenic system was not successful and widespread if it was an intermediate step toward oxygenic photosynthesis.

Finally, we must consider that over almost 3.5 billion years of photosynthesis evolution, most of the forms that ever existed are now extinct. Photosynthesis was probably a process of major significance for early life on earth and may have shaped natural environments and considerably promoted early bacterial evolution. In particular, anoxygenic anaerobic photosynthetic bacteria may have dominated the microbial world during the anoxic period of evolution. Nobody can estimate the degree of loss that has occurred over geological time scales, whether, e.g., one species out of a million or out of a billion has survived until today. In any case, the vast majority of ever-existing species have certainly gone extinct over the course of evolution, though there is still a chance that some so-far-unrecognized forms may have survived in special ecological niches but have not been identified so far. Therefore, the picture that we can draw from the available information represents only a minor part of a puzzle and we have to be aware that our view remains fragmentary.

## Data Availability

The genomic data used in this study were derived from the NCBI Genbank resources available in the public domain: https://www.ncbi.nlm.nih.gov/genbank/ (accessed on 19 February 2026). The sequences for the reaction center proteins, BchN, and AcsF were obtained from the respective genomes from the BV-BRC database, which is linked directly to the NCBI Genome database. Information on the genomic data used is presented in Table S1.

## Supplementary Materials

The following supporting information can be downloaded at https://www.mdpi.com/article/10.3390/cimb48030306/s1.

## Abbreviations

The following abbreviations are used in this manuscript:

psrc	Photosynthetic reaction center
PS	Photosystem
Bch	Bacteriochlorophyll
AcsF	Aerobic cyclisation system Fe-containing subunit
