# Supplementary material for: Photosynthesis Has Been Established Only Once—Evolution of Photosynthetic Reaction Center Proteins and Bacteriochlorophyll Biosynthesis
Source: Curr Issues Mol Biol. 2026 Mar 12;48(3):306. doi: 10.3390/cimb48030306 (PMC13025772; doi:10.3390/cimb48030306)
Supplement: Supplementary file 1 [file cimb-48-00306-s001.zip › Supplementary legends.pdf]

Figure S1: Phylogenetic tree using the type I and type II reaction center protein sequences. Sequences were translated sequences derived from the whole genome sequences. Accession numbers are indicated for each sequence. The phylogenetic tree was calculated in MEGA11 using the Maximum Likelihood method and Le_Gascuel model with Gamma distribution and allowing for some sites to be evolutionarily invariable (LG+G+I method) and iTOL was used to draw the phylogenetic tree. Bootstrap values were generated from 200 bootstrapping rounds. The tree was midpoint rooted and the different colored clades contain sequences from cyanobacteria, heliobacteria, chlorobi, chloroacido, chloroflexi and purple bacteria. This is an alternate, rectangular representation of Figure 1.

Figure S2: Synteny of the photosynthetic gene region in different bacterial groups. Bacterial groups were analyzed based on the photosynthesis gene organizational structure. The Compare Region Viewer in BV-BRC uses global PGFam families to compare a set of genes that match a focus gene and sorts the PGFams by BLAST scores. Genes are colored based on their family membership. Common to all phototrophic Alphaproteobacteria is the presence of the pucC-like gene, and of pucC associated to pufH, and in most species of the ppsR-ppaA couple, however 7 different subgroups can be separated based on the photosynthetic gene synteny: Groups **A1-A7**. The phototrophic Betaproteobacteria form 3 groups: Characteristic for group **B1**, the *Rhodocyclus* species, is the lack of ppsR-ppaA and of acsF. In group **B2**, the *Rubrivivax* species, ppsR-*ppa*A and *acs*F genes are present. In group **B3** with the *Rhodoferax* species, *pps*R, *ppa*A and *acs*F are present, and *pps*R is located at its standard place aside of *bch*G, but unusually *ppa*A is not within the usual cluster. At least five major groups of species can be distinguished among the phototrophic Gammaproteobacteria. **G1** and **G2** contain the Chromatiaceae; Group G2 is a modification of group 1 by the inclusion of *puc*C between *bch*M and *puf*H; Group G3 is represented by the species with *bchl*-b; Group **G4** is represented by *Ectothiorhodospira* species; In group **G5**, the *Halorhodospira* species, a supercluster with combined gene synteny clusters is formed. In Group **Helio**, the Heliobacteria show a few clusters relevant for photosynthesis but a higher degree of synteny is realized than in Chlorobi and Cyanobacteria.

Table S1 contains information on genome sources used for the present study.
