# Supplementary material for: Photosynthesis Has Been Established Only Once—Evolution of Photosynthetic Reaction Center Proteins and Bacteriochlorophyll Biosynthesis
Source: Curr Issues Mol Biol. 2026 Mar 12;48(3):306. doi: 10.3390/cimb48030306 (PMC13025772; doi:10.3390/cimb48030306)
Supplement: Supplementary file 1 [file cimb-48-00306-s001.zip › Table S1.pdf]

microorganisms

Table S1. Genomes (NCBI genome accession numbers) used as sources for analyses of phylogenetic analyses of *acsF*, *bchN* genes and *bchN* synteny.

AscF genes:

Afifella marina strain IM162	NHSC01000023
Anabaena variabilis ATCC 29413	NC_007413
Chloroflexus aggregans DSM 9485	NC_011831
Chloroflexus aurantiacus J-10-fl	NC_010175
Cyanothece sp. CCY0110	NZ_AAXW01000003
Cyanothece sp. CCY0110	NZ_AAXW01000065
Cyanothece sp. PCC 7424	NC_011729
Cyanothece sp. PCC 7425	NC_011884
Cyanothece sp. PCC 8801	NC_011726
Lamprocystis purpurea DSM 4197	ARBC01000023
Methylobacterium extorquens AM1	NC_012808
Microcystis aeruginosa NIES-843	NC_010296
Nostoc punctiforme PCC 73102	NC_010628
Rhodobaca barguzinensis strain DSM 19920	SODJ01000004
Rhodobacter megalophilus strain DSM 18937	FZOV01000005
Rhodobacter sphaeroides 2.4.1	CP030271
Rhodobaculum claviforme strain LMG 28126	NHSD01000320
Rhodobium orientis DSM 11290	JACIGG010000008
Rhodoblastus sphagnicola DSM 16996	JACIGC010000002
Rhodoferax antarcticus DSM 24876	CP019240
Rhodopila globiformis DSM 161	NHRY01000252
Rhodothalassium salexigens IM261	NHSB01000041
Rhodothalassium salexigens IM265	NHSA01000023
Rhodovulum imhoffii DSM 18064	NHSI01000036
Rhodovulum sulfidophilum IM796	NHRZ01000004
Roseiflexus castenholzii DSM 13941	NC_009767
Roseiflexus sp. RS-1	NC_009523
Synechococcus sp. JA-2-3B'a (2-13)	NC_007776
Synechococcus sp. PCC 7335	NZ_DS989904
Thermosynechococcus elongatus BP-1	NC_004113
Trichodesmium erythraeum IMS101	NC_008312

BchN genes:

Afifella marina IM162	NHSC01000023
Allochromatium humboldtianum DSM 21881	JABZEO010000006
Allochromatium palmeri DSM 15591	WNKT01000051

Allochromatium vinosum DSM 180	NC_013851
Allochromatium warmingii DSM 173	FNOW01000001
Anabaena variabilis ATCC 29413	NC_007413
Candidatus *Chloracidobacterium thermophilum* B	NC_016024
Chloracidobacterium sp. BV2-C	CP072648
Chloracidobacterium sp. CP2_5A	NKPT01000034
Chloracidobacterium sp. MS 40/45	CP072646
Chlorobaculum parvum NCIB 8327	NC_011027
Chlorobaculum thiosulfatiphilum DSM 249	VDCH01000023
Chlorobium chlorochromatii CaD3	NC_007514
Chlorobium ferrooxidans DSM 13031	NZ_AASE01000013
Chlorobium limicola DSM 245	NC_010803
Chlorobium phaeobacteroides DSM 266	NC_008639
Chlorobium tepidum TLS	NC_002932
Chloroflexus aggregans DSM 9485	NC_011831
Chloroflexus aurantiacus J-10-fl	NC_010175
Chloroherpeton thalassium ATCC 35110	NC_011026
Cyanothece sp. CCY0110	NZ_AAXW01000040
Cyanothece sp. PCC 7424	NC_011729
Cyanothece sp. PCC 7425	NC_011884
Cyanothece sp. PCC 8801	NC_011726
Ectothiorhodospira B14B	JAJNQM000000000
Ectothiorhodospira haloalkaliphila 9902	JAJNQR000000000
Ectothiorhodospira mobilis DSM 4180	FOUO01000002
Ectothiorhodospira shaposhnikovii DSM 243	NRSM01000015
Gemmatimonas phototrophica AP64	CP011454
Gemmatimonas sp. TET16	CP053085
Halochromatium roseum DSM 18859	NHSH01000042
Halochromatium salexigens DSM 4395	NHSF01000070
Halorhodospira abdelmalekii DSM 2110	NRRN01000026
Halorhodospira abdelmalekii DSM 2110	NRRN01000134
Halorhodospira halochloris DSM-1059	AP017372
Halorhodospira halophila SL1	NC_008789
Halorhodospira halophila IM9620	NHSE01000060
Heliobacterium gestii DSM 11169	WXEX01000005
Heliobacterium modesticaldum Ice1	NC_010337
Heliobacterium undosum DSM 13378	WXEY01000004
Lamprocystis purpurea DSM 4197	ARBC01000023
Marichromatium bheemlicum DSM 18632	JAAXKX010000003
Marichromatium gracile DSM 203	SMDC01000002
Marichromatium purpuratum 984	CP007031
Methylobacterium extorquens AM1	NC_012808
Microcystis aeruginosa NIES-843	NC_010296
Nostoc punctiforme PCC 73102	NC_010628

Pelodictyon phaeoclathratiforme BU-1	NC_011060
Phaeospirillum fulvum DSM 13234	FNWO01000005
Rhodobaca barguzinensis DSM 19920	SODJ01000004
Rhodobacter aestuarii DSM 19945	FTOG01000006
Rhodobacter capsulatus SB 1003	NC_014034
Rhodobacter megalophilus DSM 18937	FZOV01000005
Rhodobacter sphaeroides 2.4.1	CP030271
Rhodobaculum claviforme LMG 28126	NHSD01000320
Rhodobium orientis DSM 11290	JACIGG010000008
Rhodoblastus sphagnicola DSM 16996	JACIGC010000002
Rhodocyclus purpureus DSM 168	NHRX01000036
Rhodoferax antarcticus DSM 24876	CP019240
Rhodopila globiformis DSM 161	NHRY01000252
Rhodothalassium salexigens IM261	NHSB01000041
Rhodothalassium salexigens IM265	NHSA01000023
Rhodovulum imhoffii DSM 18064	NHSI01000036
Rhodovulum sulfidophilum IM796	NHRZ01000004
Roseiflexus castenholzii DSM 13941	NC_009767
Roseiflexus sp. RS-1	NC_009523
Synechococcus sp. JA-2-3B'a (2-13)	NC_007776
Synechococcus sp. PCC 7335	NZ_DS989904
Thermochromatium tepidum ATCC 43061	CP039268
Thermosynechococcus elongatus BP-1	NC_004113
Thiobaca trueperi DSM 13587	SMAO01000013
Thiocapsa bogorovii BBS	CP089309
Thiocapsa imhoffii DSM 21303	NRSD01000001
Thiocystis violascens DSM 198	CP003154
Thiohalocapsa halophila DSM 6210	NRRV01000028
Thiohalocapsa marina DSM 19078	VWXX01000040
Thiorhodococcus drewsii AZ1	AFWT01000009
Thiorhodococcus mannitoliphagus DSM 18266	JAAIJR010000002
Thiorhodococcus sp. AK35	AONC01000025
Thiorhodovibrio winogradskyi 6511	NHSO01000112
Thiospirillum jenense DSM 216	JABVCQ010000050
Trichodesmium erythraeum IMS101	NC_008312

BchN synteny genomes:

Allochromatium vinosum DSM 180	CP001896, CP001898, CP001897
Chloracidobacterium sp. BV2-C	CP072648, CP072649
Chlorobium limicola DSM 245	CP001097
Chloroflexus aggregans DSM 9485	CP001337
Ectothiorhodospira sp. BSL-9	CP011994
Halorhodospira halophila SL1	CP000544

Halothece sp. PCC 7418	CP003945.1
Heliobacterium modesticaldum Ice1	CP000930
Marichromatium purpuratum 984	AFWU00000000
Rhodobacter sphaeroides 2.4.1	CP030271, CP030272, CP030273, CP030274, CP030275, CP030276
Rhodopseudomonas palustris DSM 123	FODT00000000
Rhodospirillum rubrum ATCC 11170	CP000230, CP000231
Rhodothalassium salexigens IM261	NHSB00000000
Roseiflexus castenholzii DSM 13941	CP000804
