# Supplementary figures and images for: Photosynthesis Has Been Established Only Once—Evolution of Photosynthetic Reaction Center Proteins and Bacteriochlorophyll Biosynthesis
Source: Curr Issues Mol Biol. 2026 Mar 12;48(3):306. doi: 10.3390/cimb48030306 (PMC13025772; doi:10.3390/cimb48030306)

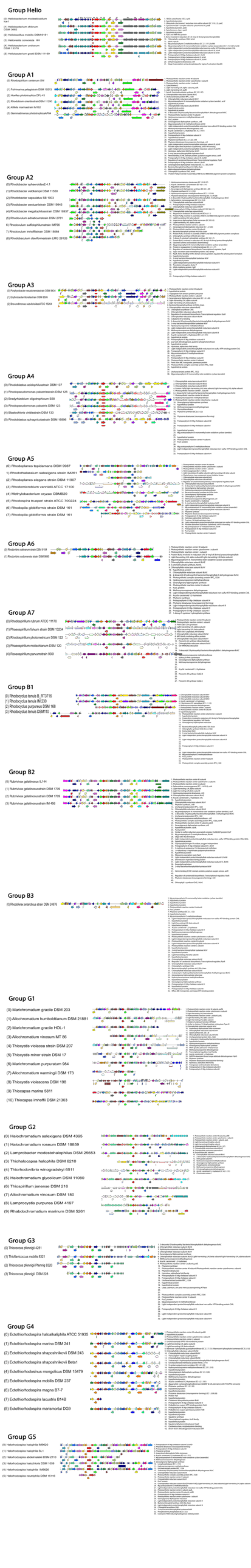

Supplement: Supplementary file 1 [file cimb-48-00306-s001.zip › FigS2_PSsynteny_combined_new2.jpg]
